# Observation of c.260A > G mutation in superoxide dismutase 1 that causes p.Asn86Ser in Iranian amyotrophic lateral sclerosis patient and absence of genotype/phenotype correlation

**Published:** 2015-07-06

**Authors:** Marzieh Khani, Afagh Alavi, Shahriar Nafissi, Elahe Elahi

**Affiliations:** 1 Department of Biology, School of Science, University of Tehran, Tehran, Iran; 2 Department of Neurology, School of Medicine, Tehran University of Medical Sciences, Tehran, Iran; 3 Department of Biology AND Department of Biotechnology, School of Science, University of Tehran, Tehran, Iran

**Keywords:** Amyotrophic Lateral Sclerosis, Genotype-Phenotype Correlation, Mutation, p.Asn86Ser, Superoxide Dismutase 1

## Abstract

**Background:** Amyotrophic lateral sclerosis (ALS) is the most common motor neuron disorder in European populations. ALS can be sporadic ALS (SALS) or familial ALS (FALS). Among 20 known ALS genes, mutations in C9orf72 and superoxide dismutase 1 (SOD1) are the most common genetic causes of the disease. Whereas C9orf72 mutations are more common in Western populations, the contribution of SOD1 to ALS in Iran is more than C9orf72. At present, a clear genotype/phenotype correlation for ALS has not been identified. We aimed to perform mutation screening of SOD1 in a newly identified Iranian FALS patient and to assess whether a genotype/phenotype correlation for the identified mutation exists.

**Methods: **The five exons of SOD1 and flanking intronic sequences of a FALS proband were screened for mutations by direct sequencing. The clinical features of the proband were assessed by a neuromuscular specialist (SN). The phenotypic presentations were compared to previously reported patients with the same mutation.

**Results: **Heterozygous c.260A > G mutation in SOD1 that causes Asn86Ser was identified in the proband. Age at onset was 34 years and site of the first presentation was in the lower extremities. Comparisons of clinical features of different ALS patients with the same mutation evidenced variable presentations.

**Conclusion:** The c.260A > G mutation in SOD1 that causes Asn86Ser appears to cause ALS with variable clinical presentations.

## Introduction

Amyotrophic lateral sclerosis (ALS) is a devastating neurodegenerative disorder characterized by wasting and weakness of limbs, bulbar, and respiratory muscles. ALS is accompanied by degeneration of motor neurons in the spinal cord, brainstem, and cortex. This progressive motor neuron degeneration usually leads to death 3-5 years after onset of the disease.^1^^-^^3^ ALS is the third most common neurodegenerative disease in countries of European descent.^4^^,^^5^ Incidence and prevalence of the disease in these countries are, respectively, 1-2 per 100,000 and 4-13 per 100,000.^6^^,^^7^ Although most cases of ALS appear sporadic (SALS), approximately 1-13% of cases are familial ALS (FALS). The familial cases most often show autosomal dominant inheritance.^   8 ^ The clinical features of ALS are variable among patients. Age at onset of symptoms from 1 to 94 years has been reported,^9^^,^^10^ and the site of onset can be either bulbar or in the limbs.^   11 ^ The rate of progression, and thus survival in the patients also varies significantly, from a few months to more than 10 years.^6^^,^^11^^-^^13^ Cause of death is usually respiratory failure.

To date, at least 20 ALS causing genes have been identified (http://alsod.iop.kcl.ac.uk/).^14^^-^^16^ Based on the functions of the genes, oxidative stress, axonal transport, vesicular transport, protein aggregation, and RNA metabolism are relevant to ALS pathology.^14^^,^^17^^,^^18^ Importantly, various ALS genes potentially have roles in the etiology of several other neurodegenerative diseases.^   15 ^ For example, mutations in the ALS gene C9orf72 have been observed in frontotemporal dementia and Parkinson’s disease patients.^   15 ^

Mutations in superoxide dismutase 1 (SOD1) and C9orf72 are the most common genetic causes of ALS, although their relative contribution varies in different populations.^12^^,^^17^^,^^19^^-^^25^ SOD1 encodes copper-zinc superoxide dismutase (Cu/Zn SOD). C9orf72 mutations are more common than SOD1 mutations in Western populations.^   4 ^ SOD1 mutations among Iranian patients are more frequent than C9orf72 mutations*,* having been observed, respectively, in approximately 12% and 2.6% of patients. The frequency of SOD1 mutations is even higher among Iranian FALS cases (38.5%).^        12 ^ Whereas SOD1 was identified as an ALS gene in 1993 and was the first gene to be identified, C9orf72 was identified only in 2011.^17^^,^^19^^,^^25^ Worldwide, more than 170 ALS causing mutations in SOD1 are reported in Human Gene Mutation Database (HGMD 2014.2; http://www.hgmd.org/).

As already stated, clinical features of ALS patients are variable. It is expected that this variation may partly be due to differences in causative gene, to different mutations in the same gene, or to variations in genetic background of individuals that carry identical mutations in the same gene. Clearly, it is expected that among these groups, there would exist least variation in the clinical features of patients with the same mutation in the same gene. For SOD1 mutations studied till now, there is generally no clear genotype/phenotype correlation for different SOD1 mutations.^   26 ^ Other words, patients with different mutations may have similar presentations and different patients with the same mutation may have different presentations. In this regard, mutations p.Ala4Val, p.Gly85Ser, p.Asp90Ala, and p.Leu144Ser may be exceptions. P.Ala4Val and p.Gly85Ser almost always cause rapidly progressive ALS.^   6 ^ p.Asp90Ala and p.Leu144Ser are associated with long survival time.^12^^,^^13^ As SOD1 mutations are relatively common among Iranian patients, we were interested to further explore genotype/phenotype correlations of SOD1 mutations. In this context, we here identified a relatively rare SOD1 mutation (c.260A > G that causes p.Asn86Ser) in an Iranian ALS family. The mutation was earlier reported in one Pakistani and one Japanese ALS family, and we aimed to the best of our ability to ascertain whether a genotype/phenotype correlation for this mutation exists by comparing the clinical features in the Iranian family to the features in the previously reported families with the same mutation.^26^^,^^27^ The genotype/phenotype correlation for this mutation was not previously investigated.

## Materials and Methods

The research was performed in accordance with the Helsinki Declaration and with approval of the Ethics Board of the University of Tehran, Iran. The patient studied agreed to participate after being informed of the nature of the research. The patient was recruited in 2014 from the Neuromuscular Clinic of Shariati Hospital, affiliated with Tehran University of Medical Sciences, where the diagnosis had been made. The clinical parts of the research were performed at the same hospital. The genetic studies were done at the College of Science of the University of Tehran.

**Figure 1:**
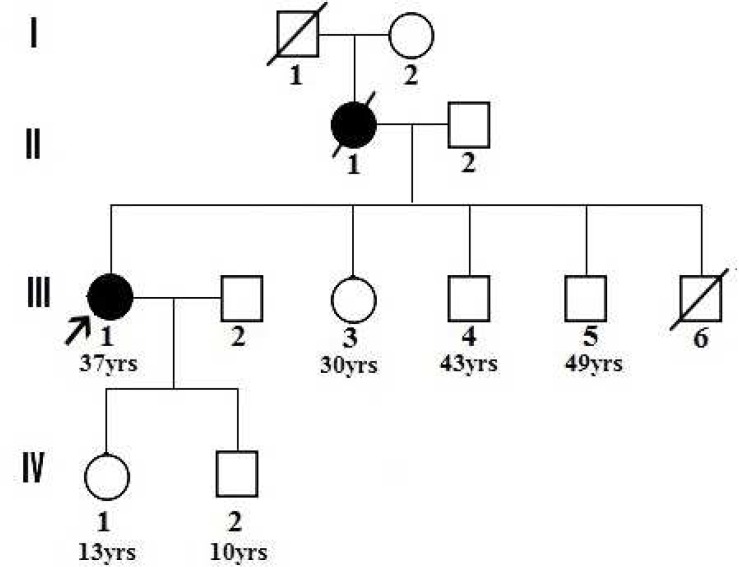
ALS187 pedigree. ▪ and ● , ALS affected individuals; □ and O , asymptomatic individuals. Arrow shows proband. Present age on some individuals is shown. Cause of death of I-1 and III-6 is unknown.

The proband of family ALS187 (III-1) was definitively diagnosed with ALS by a neuromuscular specialist (SN) according to El Escorial criteria.^   28 ^ Weakness, hyperreflexia, spasticity, progression over time, nerve conduction data, and electromyography results are among the factors included in the diagnosis protocol. According to the criteria, the involvement of at least three regions of lower and upper motor neurons allows for definitive diagnosis of ALS. The patient belonged to a small FALS pedigree that in addition to the proband included one additional ALS patient who was deceased at the time of this study (Figure 1).

Genomic DNA from peripheral blood of the proband was isolated using a standard phenol-chloroform method. The five exons of SOD1 and flanking intronic sequences were amplified by polymerase chain reactions (PCR) (Tables 1 and 2).^        12 ^ The nucleotide sequences of primers used are presented in table 3. All PCR products were subsequently sequenced with the same primers used in the PCRs, using the ABI big dye chemistry and an ABI Prism 3700 instrument (Applied Biosystems, Foster City, CA, USA). Sequences were analyzed with the Sequencher 4.10.1 software (Gene Codes Corporation, Ann Arbor, MI, USA).

SOD1 reference sequences used were NC_000021.8, NM_000454.4, and NP_000445.1. On identification of a putative disease-causing variation, evolutionary conservation of the affected amino acid was assessed by comparison to amino acid sequences of SOD1 proteins from 16 species (http://www.uniprot.org/uniprot/). The sequences were aligned using ClustalW2 software (http://www.ebi.ac.uk/Tools/msa/clustalw2/). In addition, the SIFT; (http://sift.bii.a-star.edu.sg/www/SIFT_seq_submit2.html), PolyPhen (http://genetics.bwh.harvard.edu/pph2), Panther (http://www.pantherdb.org/tools/csnpScoreForm.jsp), and SNAP ttps://rostlab.org/services/snap/submit) bioinformatics tools were used to predict the potential pathological effects of the mutation. Having identified the disease mutation, a correlation between genotype and phenotype was assessed by comparison of available clinical data on patients from three ALS families who carried the same mutation in SOD1. Two families were from Pakistan and Japan, and the third was the family from Iran reported in this study.^26^^,^^27^

## Results

***Clinical data***

The ALS patient studied here is a member of a FALS pedigree that includes two affected individuals distributed in two consecutive generations. Available clinical information on the patients is presented in table 4. Age at onset of symptoms in both was in the mid-third decade of life. Sites of earliest manifestation in II-1 and III-1 were, respectively, in the arms and legs. Whereas the mother (II-1) died 1-year after onset, her daughter is alive and relatively functional 4 years after onset. 

***Genetic analysis***

**Figure 2:**
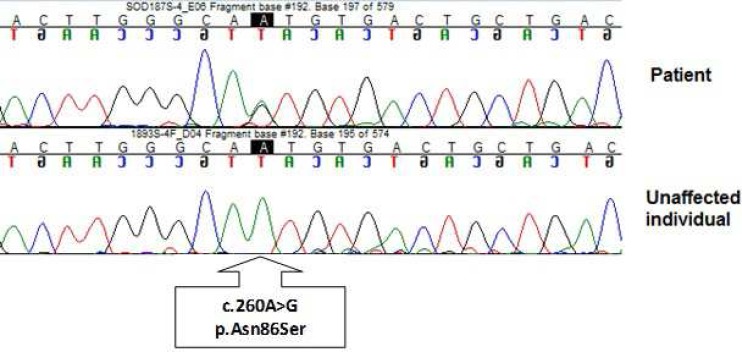
DNA sequence chromatograms showing the c.260A > G mutation and the wild-type sequence. The mutation that causes p.Asn86Ser is evident in the in the heterozygous state in the chromatogram of the proband

Heterozygous mutation c.260A > G that causes p.Asn86Ser in the encoded SOD1 protein was observed in the DNA of the proband (Figure 2).

**Table 1:** Polymerase chain reactions** (**PCR) conditions for five exons (E1-E5) of superoxide dismutase 1 (SOD1)

**PCR ingredients**	**E1**	**E2**	**E3**	**E4**	**E5**
Buffer (×10) (µl)	2.0	2.0	2.0	2.0	2.0
MgCl_2_ (mM)	1.0	1.0	1.0	1.0	1.5
dNTP (mM)	0.2	0.2	0.2	0.2	0.3
Primer F (pM)	5.0	5.0	5.0	5.0	6.0
Primer R (pM)	5.	5.0	5.0	5.0	6.0
DNA template (ng)	150.0	150.0	150.0	150.0	200.0
Taq polymerase (unit)	1.0	1.0	1.0	1.0	1.0
DMSO (%)	7.50%	-	-	-	-
Betaine (M)	-	-	-	1.0	-
ddH_2_O	13.0	13.0	13.0	13.0	12.0
Total volume	20.0	20.0	20.0	20.0	20.0

PCR: Polymerase chain reactions, dNTP: Deoxynucleotide triphosphates

**Table 2:** Thermocycler conditions for polymerase chain reactions (PCR) of five exons (E1-E5) of superoxide dismutase 1 (SOD1)

**Cycling steps**	**E1**	**E2**	**E3**	**E4**	**E5**
Initial denaturation	94 °C/5 min	94 °C/5 min	94 °C/5 min	94 °C/5 min	94 °C/5 min
Denaturation	94 °C/1 min	94 °C/1 min	94 °C/1 min	94 °C/1 min	94 °C/1 min
Annealing	61 °C/50 s	62 °C/50 s	63 °C/50 s	62 °C/40 s	58 °C/1 min
Extension	72 °C/50 s	72 °C/50 s	72 °C/50 s	72 °C/50 s	72 °C/50 s
Final extension	72 °C/5 min	72 °C/5 min	72 °C/5 min	72 °C/5 min	72 °C/5 min
Number of cycles	35	35	35	35	35

PCR: Polymerase chain reactions, SOD1: Superoxide dismutase 1

**Table 3:** The nucleotide sequences of primers.

**Primer name**	**Sequence 5** **ʹ** ** to 3** **ʹ**	**Primer ** **name**	**Sequence 5** **ʹ** ** to 3** **ʹ**	**Product ** **Size (bp)**	
SOD1-1F	GTTCTGGACGTTTCCCGGCTG	SOD1-1R	GTGACTCAGCACTTGGGCACC	542	
SOD1-2F	AGAGCAGTTAAGCAGCTTGCTG	SOD1-2R	CATGAGGATCAATGGAGCCTG	477	
SOD1-3F	TCACTGTGGCTGTACCAAGGTG	SOD1-3R	CCAGGAAGTAAAAGCATTCCAGC		394
SOD1-4F	CCAGAGCATTAGTGTGTAGACG	SOD1-4R	TGAGAAACCCAATCCTGGCAAG	600	
SOD1-5F	AGGTAATGTCTTTGCAACACCAAG	SOD1-5R	CCTATTTGTCTAAGCAGAGTTGTG		826

SOD1: Superoxide dismutase 1

**Table 4:** Description of Amyotrophic lateral sclerosis (ALS) diagnosed individuals with p.Asn86Ser mutation in superoxide dismutase 1 (SOD1)

**Patient**	**Iranian**		**Pakistani**		**Japanese**	
ID	II-1	III-1	patient 1α	patient 2α	patient 1β	patient 2β
Sex	Female	Female	Female	Male	Male	Female
Present age	Dead	38 year	Dead	Dead	Dead	36 year
Age at onset (year)	37	34	13	33	52	34
Survival time	1 year	> 4 year Δ	14 week	11 month	4 year	> 11 year
Site of earliest manifestation	Arms	Legs	Legs	-	Upper body	Lower limb
Genotype*	wt/mut**	wt/mut	mut/mut	wt/mut**	wt/mut	wt/mut

* wt: wild type allele; mut: p.Asn86Ser;

** inferred genotype;

Δ : still alive;

α , belong to same Pakistani family;

β , belong to same Japanese family

No additional variation was detected. Segregation analysis in the pedigree was not possible because the only other affected individual in the pedigree (II-1) was deceased and the living asymptomatic members of the pedigree did not want to know whether or not they carried the mutated allele. However, an asparagine at positions corresponding to p.86 in the human SOD1 protein is highly conserved across species from Caenorhabditis elegans to Homo sapiens (Table 5). The SIFT, PolyPhen, Panther, and SNAP tools predicted, respectively, that the substitution is deleterious, probably damaging, deleterious, and non-neutral. Finally, the same variation was previously reported as a cause of ALS in two families, one from Pakistan^26^ and the other from Japan.^27^ The sum of these data allowed us to conclude that the p.Asn86Ser causing variation in SOD1 was the probable cause of ALS in the proband and her affected mother. The inheritance pattern of ALS in the pedigree appears to be autosomal dominant, consistent with observation of a single mutated allele in the proband who was born to non-consanguineous parents and whose mother was also affected (Figure 1).

## Discussion

In the present study, we identified a mutation in SOD1 that causes p.Asn86Ser in the proband of Iranian FALS pedigree ALS187. ALS inheritance in the pedigree was autosomal dominant, without evidence of anticipation. The p.Asn86Ser mutation was twice reported previously, once in a Pakistani pedigree and once in a Japanese pedigree.^26^^,^^27^ Comparisons of phenotypic features among patients of Iranian, Pakistani, and Japanese origin reveal notable intra-familial and especially interfamilial variability in disease presentation (Table 4). Age at onset of symptoms ranged from 13 to 52 years among the six patients of the three pedigrees. Age at onset was similar for the two patients of the Iranian pedigree, but it differed by about 20 years in the patients of both the Pakistani and the Japanese families. Although the very early onset at the age of 13 years in one of the Pakistani patients may be partly due to the homozygous status of her mutated genotype, both patients in the Japanese family were heterozygotes. The two affected individuals in the Pakistani family had a niece-uncle relationship. The parents of the affected 13-year-old child, who are presumably obligate carriers, were reported to be asymptomatic in the third decade of their lives. Survival time was similarly variable among the patients, with three surviving < 1-year after onset and three surviving for over 4 years. Survival time differed by at least 7 years in the two patients of the Japanese family. The earliest manifestation was in the limbs for all five patients with available data, but in the lower limbs in three and in the upper limbs in two. In regard to site of earliest manifestation, there was intrafamilial variation in the Iranian and in the Japanese families. In all the families, the earliest presentation was asymmetric. Furthermore, known cause of death was respiratory failure in all four deceased individuals. Taken together, the data emphasize the absence of a tight genotype/phenotype correlation for the p.Asn86Ser mutation in SOD1. Clearly, variability in expression may be due to differences in genetic backgrounds, to environmental factors, or to stochastic events during development. The existence of multiple ALS causing genes begs the consideration of whether these genes can have collective or modifying effects. Specifically, it is possible that even polymorphisms in other ALS causing genes will affect subtle features of disease presentation in patients with the p.Asn86Ser mutation in SOD1. Lack of tight association between genotype and phenotype renders counseling and prognosis problematic.

Other than the p.Asn86Ser mutation, the only other SOD1 mutations ever reported in the homozygous state in ALS patients are p.Leu84Phe, p.Asp90Ala, and p.Leu117Val.^10^^,^^12^^,^^29^ The sum of data in families harboring these mutations do not definitively show that mutations in the homozygous state result in a more severe phenotype. With respect to p.Asn86Ser, the extent of phenotypic variation between the homozygote and heterozygote patients in the Pakistani family may be comparable to the extent of variation between the two Japanese patients who are heterozygous carriers. It appears that the consequences of the p.Asn86Ser mutation in SOD1 is not strictly uniform with respect to age at onset, site of presentation, and duration of the disease irrespective of being in the homozygous or heterozygous state.

**Table 5:** Conservation of p.Asn86 in superoxide dismutase 1 (SOD1) proteins

**Organism**	**Seq ID**	**Amino acid sequence** *
Homo sapiens	P00441	RHVGDLG**N**VTADKDGVA
Pan troglodytes	P60052	RHVGDLG**N**VTADKDGVA
Macaca mulatta	Q8HXQ0	RHVGDLG**N**VTAGKDGVA
Bos taurus	P00442	RHVGDLG**N**VTADKNGVA
Equus caballus	P00443	RHVGDLG**N**VTADENGKA
Cavia porcellus	P33431	RHVGDLG**N**VTAGADGVA
Sus scrofa	P04178	RHVGDLG**N**VTAGKDGVA
Ovis aries	P09670	RHVGDLG**N**VKADKNGVA
Canis familiaris	Q8WNN6	RHVGDLG**N**VTAGKDGVA
Oryctolagus cuniculus	P09212	RHVGDLG**N**VTAGSNGVA
Rattus norvegicus	P07632	RHVGDLG**N**VAAGKDGVA
Mus musculus	P08228	RHVGDLG**N**VTAGKDGVA
Gallus gallus	P80566	RHVGDLG**N**VTA-KGGVA
Prionace glauca	P11418	RHVGDLG**N**VEANGNGVA
Xiphias gladius	P03946	RHVGDLG**N**VTADANGVA
Caenorhabditis elegans	P34697	RHVGDLG**N**VEAGADGVA

Seq ID: Sequence ID numbers at the Uniprot server;

* Position of amino acid change is shown in bold

## Conclusion

The p.Asn86Ser mutation in SOD1 appears to cause disease with variable clinical presentations. There is no clear genotype/phenotype correlation for the p.Asn86Ser mutation; the clinical phenotype associated with this mutation may be influenced by the genetic background of the patient and possibly by environmental factors.

## Notes:

**How to cite this article: **Khani M, Alavi A, Nafissi Sh, Elahi E. Observation of c.260A > G mutation in superoxide dismutase 1 that causes p.Asn86Ser in Iranian amyotrophic lateral sclerosis patient and absence of genotype/ phenotype correlation. Iran J Neurol 2015; 14(3): 152-7.
